# Supplementary material for: Hybrid Models and Biological Model Reduction with PyDSTool
Source: PLoS Comput Biol. 2012 Aug 9;8(8):e1002628. doi: 10.1371/journal.pcbi.1002628 (PMC3415397; doi:10.1371/journal.pcbi.1002628)
Supplement: Text S4 — Complete source code for the PyDSTool package (version 0.88.120504). Includes API documentation and help files linking to web pages. This file is identical to the current public release on Sourceforge.net. (ZIP) [file pcbi.1002628.s004.zip › PyDSTool/html/PyDSTool.Toolbox.optimizers.helpers-module.html]

xml version="1.0" encoding="ascii"?


PyDSTool.Toolbox.optimizers.helpers


| Home | Trees | Indices | Help | PyDSTool |
| --- | --- | --- | --- | --- |

Package PyDSTool ::
Package Toolbox ::
Package optimizers ::
Package helpers

[hide private]

[frames] | no frames]

# Package helpers

source code

Helper functions

Fitting functions :

- Quadratic defines a quadratic cost

NB : the first dimension of the cost, gradient or hessian is the
number of points to fit, the second is the dimension of the point if
there is one. This leads to the fact that the gradient returns in fact
the jacobian of the function.

Finite Difference functions :

- ForwardFiniteDifferences
- CenteredFiniteDifferences
- also, versions with in-built caching of previous values


|  |  |
| --- | --- |
| Submodules | [hide private] |

- **PyDSTool.Toolbox.optimizers.helpers.finite\_difference**
- **PyDSTool.Toolbox.optimizers.helpers.levenberg\_marquardt**
- **PyDSTool.Toolbox.optimizers.helpers.quadratic**

  


|  |  |
| --- | --- |
| Classes | [hide private] |

Quadratic  
Defines a cost function with a quadratic cost

CenteredFiniteDifferences  
A function that will be able to computes its derivatives with a
centered difference formula

ForwardFiniteDifferencesCache  
A function that will be able to computes its derivatives with a
forward difference formula.

ForwardFiniteDifferences  
A function that will be able to computes its derivatives with a
forward difference formula

FiniteDifferencesFunction


|  |  |
| --- | --- |
| Variables | [hide private] |

helpers\_\_all\_\_ = `['Quadratic', 'FiniteDifferencesFunction', 'F...`


|  |  |
| --- | --- |
| Variables Details | [hide private] |

### helpers\_\_all\_\_

Value:
:   ```
    ['Quadratic',
     'FiniteDifferencesFunction',
     'ForwardFiniteDifferences',
     'CenteredFiniteDifferences',
     'ForwardFiniteDifferencesCache']
    ```

  


| Home | Trees | Indices | Help | PyDSTool |
| --- | --- | --- | --- | --- |

|  |  |
| --- | --- |
| Generated by Epydoc 3.0.1 on Fri May 4 15:24:05 2012 | http://epydoc.sourceforge.net |
